# Cardiothoracic ratio values and trajectories are associated with risk of requiring dialysis and mortality in chronic kidney disease

**DOI:** 10.1038/s43856-023-00241-9

**Published:** 2023-02-07

**Authors:** Che-Yi Chou, Charles C. N. Wang, Hsiu-Yin Chiang, Chien-Fong Huang, Ya-Luan Hsiao, Chuan-Hu Sun, Chun-Sheng Hu, Min-Yen Wu, Sheng-Hsuan Chen, Chun-Min Chang, Yu-Ting Lin, Jie-Sian Wang, Yu-Cuyan Hong, I-Wen Ting, Hung-Chieh Yeh, Chin-Chi Kuo

**Affiliations:** 1 Division of Nephrology, Department of Internal Medicine, Asia University Hospital, Wufeng, Taichung, Taiwan; 2 Department of Post-baccalaureate Veterinary Medicine, Asia University, Wufeng, Taichung, Taiwan; 3 Division of Nephrology, Department of Internal Medicine, China Medical University Hospital and College of Medicine, China Medical University, Taichung, Taiwan; 4 Department of Bioinformatics and Medical Engineering, Asia University, Taichung, Taiwan; 5 Big Data Center, China Medical University Hospital and College of Medicine, China Medical University, Taichung, Taiwan; 6 Department of Health Administration, Johns Hopkins Bloomberg School of Public Health, Johns Hopkins University, Baltimore, MD USA; 7 Department of Electrical and Computer Engineering, University of Wisconsin-Madison, Madison, WI USA; 8 AKI-CARE (Clinical Advancement, Research and Education) Center, Department of Internal Medicine, China Medical University Hospital and College of Medicine, China Medical University, Taichung, Taiwan

**Keywords:** Nephrology, Chronic kidney disease

## Abstract

**Background:**

The prognostic role of the cardiothoracic ratio (CTR) in chronic kidney disease (CKD) remains undetermined.

**Methods:**

We conducted a retrospective cohort study of 3117 patients with CKD aged 18–89 years who participated in an Advanced CKD Care Program in Taiwan between 2003 and 2017 with a median follow up of 1.3(0.7–2.5) and 3.3(1.8–5.3) (IQR) years for outcome of end-stage renal disease (ESRD) and overall death, respectively. We developed a machine learning (ML)–based algorithm to calculate the baseline and serial CTRs, which were then used to classify patients into trajectory groups based on latent class mixed modelling. Association and discrimination were evaluated using multivariable Cox proportional hazards regression analyses and C-statistics, respectively.

**Results:**

The median (interquartile range) age of 3117 patients is 69.5 (59.2–77.4) years. We create 3 CTR trajectory groups (low [30.1%], medium [48.1%], and high [21.8%]) for the 2474 patients with at least 2 CTR measurements. The adjusted hazard ratios for ESRD, cardiovascular mortality, and all-cause mortality in patients with baseline CTRs ≥0.57 (vs CTRs <0.47) are 1.35 (95% confidence interval, 1.06–1.72), 2.89 (1.78–4.71), and 1.50 (1.22–1.83), respectively. Similarly, greater effect sizes, particularly for cardiovascular mortality, are observed for high (vs low) CTR trajectories. Compared with a reference model, one with CTR as a continuous variable yields significantly higher C-statistics of 0.719 (vs 0.698, *P* = 0.04) for cardiovascular mortality and 0.697 (vs 0.693, *P* < 0.001) for all-cause mortality.

**Conclusions:**

Our findings support the real-world prognostic value of the CTR, as calculated by a ML annotation tool, in CKD. Our research presents a methodological foundation for using machine learning to improve cardioprotection among patients with CKD.

## Abstract (web summary)

Chou et al. develop a machine learning algorithm for the automated determination of cardiothoracic ratio (CTR). Both baseline CTRs and trajectories of serial CTR measurements are associated with end-stage renal disease and mortality in patients with chronic kidney disease.

## Plain language summary

An enlarged heart occurs during various medical conditions and can result in early death. However, it is unclear whether this is also the case in patients with chronic kidney disease (CKD). Although the size of the heart can be measured on chest X-rays, this process is time consuming. We used artificial intelligence to quantify the heart size of 3117 CKD patients based on their chest X-rays within hours. We found that CKD patients with an enlarged heart were more likely to develop end-stage kidney disease or die. This could improve monitoring of CKD patients with an enlarged heart and improve their care.

## Introduction

The cardiothoracic ratio (CTR) was first defined in 1919 and has since been accepted as a means of quantifying heart size and volume on posterior-anterior chest X-rays (PA-CXRs)^1,2^. A CTR of <0.5 is considered normal, whereas a CTR >0.5 indicates cardiomegaly. The CTR is a key prognostic indicator in many conditions, such as coronary artery disease^3^, end-stage renal disease (ESRD)^4,5^, and conditions related to aging^6^. A higher CTR value is generally associated with poor prognosis, and 2 longitudinal studies of healthy populations have reported significant associations between high CTR values and cardiovascular (CV) mortality^7,8^. Several studies have challenged the current CTR threshold; their results have revealed CTRs below 0.5 to be associated with increased CV mortality in the general population^7,9^. Zaman et al. observed that a preangiographic CTR above 0.42 to be associated with increased all-cause mortality^3^.

Studies have demonstrated that the CTR is a significant indicator of mortality and deteriorating functional status for patients with incident dialysis^10,11^ and ESRD requiring regular hemodialysis^4,12–14^ or peritoneal dialysis^5^. In addition, the CTR has been used to monitor fluid overload, malnutrition, and heart disease in patients receiving dialysis^4,10,12^. However, the independent value of the CTR for predicting disease progression and mortality among patients with nondialysis chronic kidney disease (CKD-ND) is unclear. A recent study reported a CTR > 0.5 and the presence of aortic arch calcification are associated with rapid renal progression as well as CV and all-cause mortality in CKD-ND^15^. However, the researchers could not assess the risk of progression to dialysis or determine an optimal risk cutoff for the CTR because the few patients enrolled in the study and the short follow-up. To date, much of the evidence on the role of the CTR in ESRD originates from studies conducted in Taiwan because semiannual CTR measurement is required by the Taiwan Society of Nephrology for evaluation of dialysis adequacy. Nevertheless, the CTR is not considered in the routine care of the Advanced CKD Care Program in Taiwan or similar programs in other countries; this may partially explain the limited availability of related data in CKD-ND. Furthermore, despite ongoing efforts to investigate the prognostic role of the CTR in various disease states, the validity and generalizability of these studies are uncertain because sample sizes are often small; CTR measurement requires labor-intensive annotation by physicians. Although several machine learning (ML) algorithms have been developed to simplify and standardize the annotation process, adoption of these algorithms has been slow because the algorithms require further validation before they can be fully integrated into the clinical workflow^16–20^. To address this research gap and evaluate the risks of requiring dialysis and mortality associated with the various baseline CTRs and CTR trajectories in patients with CKD-ND, we developed an artificially intelligent CTR (iCTR) assessment system to annotate PA-CXRs. We further evaluated whether the incorporation of the CTR into the workflow could improve the predictive performance of the Kidney Failure Risk Equation (KFRE) and a conventional mortality risk model for CKD^21,22^. Our findings identify the positive associations between CTR and accelerated progression to ESRD or mortality in CKD and demonstrate the real-world prognostic value of CTR in predicting mortality.

## Methods

### Study patients and design

Taiwan’s National Health Insurance launched the Project of Integrated Care of CKD in 2002. The project initially targeted patients with an estimated glomerular filtration rate (eGFR) of <60 mL/min/1.73 m^2^ or with proteinuria (urine protein-to-creatinine ratio [uPCR] > 1 [g/g]). In 2007, the project adopted a multidisciplinary approach and focused on CKD stages 3b to 5. China Medical University Hospital (CMUH), a tertiary medical center in central Taiwan, joined this care project in 2003 and prospectively enrolled consecutive patients with CKD who agreed to participate in this Advanced CKD Care Program. The details of the program were described in a previous study^23^. Biochemical markers of renal injury, including serum creatinine (S-Cre), blood urea nitrogen, and spot uPCR, were measured at least once every 3 months. In the present study, we further integrated the data from the Advanced CKD Care Program with data from the CMUH Clinical Research Data Repository, including data on laboratory tests, medications, special procedures, and admission records^24–26^. The index date was the date of patient enrollment in the Advanced CKD Care Program. All enrolled patients were followed up until the initiation of long-term renal replacement therapy (hemodialysis, peritoneal dialysis, or transplantation), death, loss to follow-up, or December 31^st^, 2019, whichever occurred first. Dates of death were verified at the National Death Registry of the Ministry of Health and Welfare of Taiwan^25,26^.

**Fig. 1:**
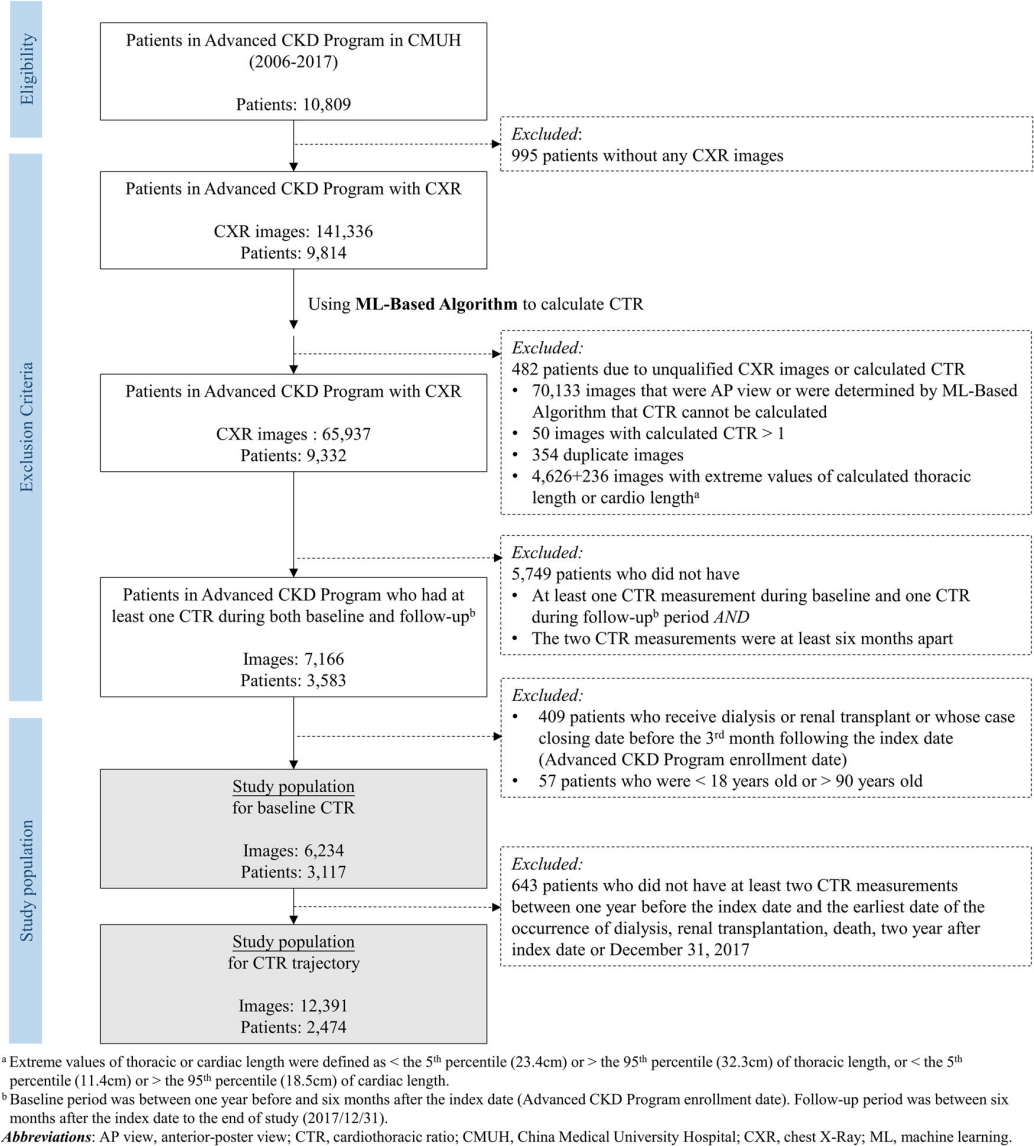
Flow diagram for included patients. Patients and chest X-ray images are identified through deep data cleaning process to form the final study population.

We included the data of patients aged 18–89 years participating in the Advanced CKD Care Program from January 1^st^, 2003, to December 31^st^, 2017, who had no history of dialysis. For population of baseline CTR analysis, we included patients who had at least one CTR measurement during the baseline period (−1 year to +6 months of the index date) and one CTR during the follow-up period (+6 months of the index date to December 31^st^, 2017), with the two CTR measurements were at least 6 months apart. Baseline CTR was the CTR in the baseline period and closest to the index date. In total, 3117 patients with 6234 CTR records were included for analysis (Fig. 1). All CTR records were derived from PA-CXRs, which were taken with the patients in upright position. The average number of days between the dates of baseline CTR and serum creatinine (S-Cre) was 83.0 (IQR 46.0–131.0). The frequency of repeated CTR measurements for the longitudinal arm was shown in the Supplementary Table 1. For population of CTR trajectory analysis, we further excluded patients who did not have at least two CTR measurements within the 2 years after the index date and a total of 2474 participants with 12391 CTR records were included in the trajectory analysis (Fig. 1). This study was approved by the Big Data Center of CMUH and the Research Ethics Committee and Institutional Review Board (REC/IRB) of CMUH, and informed consent was waived due to retrospective nature of the study (CMUH105-REC3-068 & CMUH108-REC2-022). All methods were performed in accordance with the relevant guidelines and regulations of the REC/IRB.

### Automatic determination of CTR using deep learning

We used deep learning to perform automatic cardiothoracic cavity segmentation in PA-CXR images by using a U-Net model^27^. The U-Net architecture is illustrated in Supplementary Fig. 1^27^. The model consists of a contracting path (left side) and an expanding path (right side). The contracting path follows the typical architecture of a convolutional network, consisting of repeated application of two 3 × 3 convolutions (unpadded convolutions), each of which is followed by a rectified linear unit (ReLU)^28^ function and a 2 × 2 max-pooling operation with a stride of 2 for downsampling. In each downsampling step, the number of feature channels is doubled. Each step in the expanding path consists of upsampling of the feature map followed by a 2 × 2 convolution (“up-convolution”) that halves the number of feature channels, concatenation with a correspondingly cropped feature map from the contracting path, and two 3 × 3 convolutions, each of which is followed by a ReLU function. The cropping is necessary because of the loss of border pixels with each convolution. In the final layer, a 1 × 1 convolution is used to map each 32-component feature vector to the desired number of classes. In total, the network has 23 convolutional layers. To evaluate the performance of the model, we evaluated CTR agreement as an absolute difference (AD) of <0.03 between the ML-estimated CTR and one calculated through physician annotation. From the testing database, 510 of 537 PA-CXRs had a CTR AD < 0.03, for an accuracy of 94.97%. The iCTR Assessment System that incorporates the CTR estimation algorithm has received premarket approval for software as a medical device (SaMD) from the US Food and Drug Administration (FDA; 510(k) number, K212624) and Taiwan FDA (TFDA; medical device license number, 007443). The ML-estimated CTR of actual cases were illustrated in Supplementary Fig. 2.

### Determination of kidney function

S-Cre levels were measured using the Jaffe rate method (kinetic alkaline picrate) at the CMUH central laboratory by using a Beckman UniCel DxC 800 immunoassay system (Beckman Coulter, Brea, CA, USA). eGFRs were calculated using the Chronic Kidney Disease Epidemiology Collaboration creatinine equation^29^. The S-Cre level at enrollment was used to determine the baseline eGFR and corresponding CKD stage according to the following cutoff values: eGFRs of >90, 60 to 89.9, 45 to 59.9, 30 to 44.9, 15 to 29.9, and <15 mL/min/1.73 m^2^ were respectively considered stages 1, 2, 3a, 3b, 4, and 5. Proteinuria was defined as a uPCR of >0.5 [g/g cre]. For patients with only a urine albumin-to-creatinine ratio (uACR) available, we converted the uACR into uPCR by using the following equation derived from a Japanese study: ${{{{\mathrm{ln}}}}}\left({{{{{\rm{ACR}}}}}}\right)=1.32* {{{{\mathrm{ln}}}}}\left({{{{{\rm{PCR}}}}}}\right)-2.64$^30^.

### Statistical analyses

Continuous variables were compared using the Wilcoxon rank sum test and are expressed as medians with IQRs. Categorical variables were compared using the chi-square test and are expressed as frequencies with percentages. The baseline CTR was categorized into quartiles. To characterize the trajectories of all CTR measurements among patients in the study population for the trajectory analysis, Latent Class Mixed Modelling (LCMM) was used to fit a semiparametric mixture model to the longitudinal data using the maximum likelihood method^31^. The LCMM package (version 1.9.3) in R was applied^32^. This approach is appropriate when the number of subgroups or other information, such as the trajectory shape of each subgroup, is unknown. We empirically compared 2-, 3- and 4-group solutions and optimized the number of subgroups by using Bayesian information criterion values (with a number close to 0 indicating a good fit); the trajectory shapes were determined by the order of the polynomial (e.g., linear, quadratic, or cubic). The CTR trajectories were determined before the risk analysis for dialysis and mortality.

For time-to-event analysis, the number of person-years free from dialysis and mortality after enrollment in the Advanced CKD Program was computed. We evaluated the prospective associations of both the baseline CTR and longitudinal CTR trajectories with dialysis initiation and mortality by using multiple Cox proportional hazards models. The models were adjusted based on a priori knowledge to control potential confounding variables. To characterize the dialysis risk associated with the exposures of interest, we performed a competing risk analysis using cause-specific Cox proportional hazards modeling, with deaths considered censoring events to minimize the potential bias introduced by a competing death risk. Model fit was evaluated using the Akaike information criterion. Because data were missing for some explanatory variables (eg, 15.4% missing for pooled uPCR; Supplementary Data 1), we further performed multiple imputation by using a fully conditional method in SAS, namely, an iterative Markov chain Monte Carlo procedure, for the missing variables. We set the number of imputations to 20 and the number of iterations to 100. To explore the potential effect modifiers in the association between both the baseline and longitudinal CTRs and the main outcomes, we stratified patients based on age (older or younger than 65 years), sex, hypertension, diabetes, CV disease (CVD) status, CKD stage (3 vs 4 or 5), and uPCR (> vs ≤0.5 [g/g cre]) at baseline. To evaluate the predictive value of the CTR, we compared the Uno’s C-statistics from the reference KFRE, which considers age, sex, eGFR, and uPCR (log-transformed), with that from the same model with the addition of the CTR as a categorical or continuous variable^33^. The reference mortality predictive model (RMPM) considered age, sex, eGFR, diabetes, hypertension, and anemia (hemoglobin < 12 g/dL)^21^. We also plotted the observed versus the predicted risk to reveal the differences in the calibration of the three prediction models. All statistical analyses were performed using SAS version 9.4 (SAS Institute, Cary, NC, USA) and R version 3.5.1 (R Foundation for Statistical Computing, Vienna, Austria). Statistical significance was defined as a 2-tailed *P-*value of <0.05.

### Reporting summary

Further information on research design is available in the Nature Portfolio Reporting Summary linked to this article.

## Results

### Clinical characteristics by baseline CTR quartile

The median (IQR) age at enrollment for all 3117 patients was 69.5 (59.2–77.4) years; the median duration of follow-up was 1.3 (0.7–2.5) and 3.3 (1.8–5.3) (IQR) years for ESRD events and death, respectively, as events usually occurred before death. Increasing trends in age, body mass index, and the proportions of female sex and of DM, hypertension, CVD, or stage 4 or 5 CKD at baseline were observed across the increasing quartiles of CTR (Supplementary Data 2). By contrast, the proportions of patients who were active smokers, were alcohol drinkers, and had total education of ≥16 years decreased with CTR quartile. Compared with patients with a CTR of <0.47, patients with a CTR of ≥0.57 were more likely to use antiplatelet agents and had higher baseline systolic blood pressure (median: 142.0 [123.6–158.0] vs 132.5 [119.0–148.0] mmHg) and lower baseline diastolic blood pressure (median: 73.8 [65.7–81.2] vs 76.3 [70.0–86.0] mmHg). Significant decreasing trends in baseline eGFR, hemoglobin, and urinary creatinine levels were concomitant with increasing trends in proteinuria and the baseline serum levels of phosphorus, blood urea nitrogen, and uric acid (Supplementary Data 1). In the sensitivity analysis of matching CTR values with available echocardiographic parameters such as ejection fraction, left ventricular mass index (LVMI), and left atrial volume index (LAVI), the increasing trends of LVMI, and LAVI were concordant with the increasing baseline CTR groups and CTR trajectory group (Supplementary Table 2). Detailed information about the CMUH echocardiographic cohort has been published previously^24,34^.

### Clinical characteristics by CTR trajectory

Among 2474 patients with at least 2 CTR measurements, the median (IQR) number of CTR measurements was 4.0 (2.0–6.0). We identified 3 CTR trajectories through LCMM and subdivided patients into low- (30.1%), medium- (48.1%), and high-(21.8%) trajectory groups (Supplementary Fig. 3). The clinicodemographic and laboratory trends from the low to high trajectory were similar to those observed across the baseline CTR quartiles (Supplementary Data 3). The coefficient of variation in serial CTR measurements was the greatest among patients with medium CTR trajectories. When the change in the variation of the CTR was defined using the absolute difference (AD), a significant increasing trend in AD was observed with CTR trajectory from low to high. However, other variation indicators, including slope and percentage change, were not significantly different among the CTR trajectory groups (Supplementary Data 3).

### Associations of baseline CTR and longitudinal CTR trajectories with ESRD and mortality

**Fig. 2:**
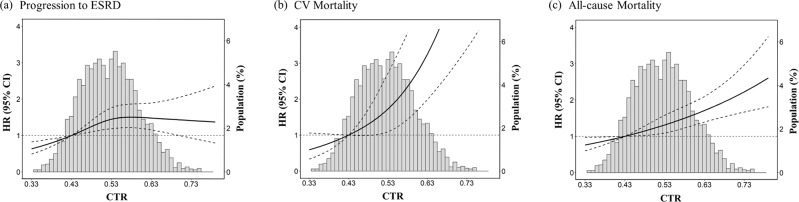
Dose-response relationship between baseline CTR and risk of developing outcomes of interest based on the study population of 3117 patients. Adjusted HRs for (**a**) progression to ESRD, (**b**) CV mortality, and (**c**) all-cause mortality based on baseline CTR. Solid black lines represent adjusted HRs based on restricted cubic splines for baseline CTR with knots at the 10th, 50th, and 90th percentiles. Dashed black lines represent 95% CI. The reference is set at the 10th percentile of baseline CTR.

In the fully adjusted model, each 10% increase in baseline CTR was significantly associated with a higher hazard ratio (HR) for progression to ESRD (adjusted HR [aHR] 1.15, 95% CI, 1.03–1.28), CV mortality (1.83, 95% CI, 1.49–2.24), and all-cause mortality (1.27, 95% CI, 1.16–1.40), respectively (Supplementary Data 4). The aHRs for progression to ESRD, CV mortality, and all-cause mortality in patients with a baseline CTR of ≥0.57 were 1.35 (95% CI, 1.06–1.72), 2.89 (95% CI, 1.78–4.71), and 1.50 (95% CI, 1.22–1.83), respectively, relative to patients with a CTR of <0.47. Furthermore, compared with the low-trajectory group, the aHRs for progression to ESRD, CV mortality, and all-cause mortality were 1.70 (95% CI, 1.28–2.25), 3.84 (95% CI, 2.28–6.45), and 1.78 (95% CI, 1.44–2.22), respectively, in the high-trajectory group (Supplementary Data 4). The dose-response relationship between ESRD and baseline CTR was curvilinear up to a CTR of 0.6, at which it plateaued (Fig. 2a). By contrast, a linear nonthreshold dose-response relationship of baseline CTR with CV and all-cause mortality was observed beyond the ratios of 0.47 and 0.50, respectively (Fig. 2b and c). In subgroup analysis for baseline CTR, patients without hypertension and advanced CKD stage 4–5 were more vulnerable to increased baseline CTR for the outcome of progression to ESRD (Supplementary Data 5). By contrast, the associations between baseline CTR and CV- or all-cause mortality were not modified by age, sex, hypertension, diabetes, CVD, CKD stage, and proteinuria (Supplementary Data 5). In subgroup analysis for CTR trajectory, female and hypertension-free patients were more susceptible to high CTR trajectory regarding the dialysis outcome (Supplementary Data 6). Patients without hypertension were also more vulnerable to CV mortality (Supplementary Data 6).

### Performance of baseline CTR in predicting dialysis and mortality

Adding the baseline CTR to the conventional 4-variable KFRE for predicting ESRD did not significantly improve the predictive performance of the KFRE (C-statistics of 0.842 to 0.843 for baseline CTR categories, *P* = 0.60 with similar calibration) (Table 1 and Supplementary Fig. 4). However, when a reference model comprising age, sex, eGFR, diabetes, hypertension, and anemia status was used for predicting mortality outcomes, incorporating baseline CTR as a continuous variable significantly increased the C-statistics from 0.698 to 0.719 (*P* = 0.04) for CV mortality (Table 1) and from 0.693 to 0.697 (*P* < 0.001) for all-cause mortality (Table 1). Integrating baseline CTR also improved the calibration performance for the outcome of CV mortality (Supplementary Fig. 4).

**Table 1:** C-statistics from KFRE and Reference Mortality Predictive Model (RMPM) with continuous or categorical CTR variable for predicting ESRD, CV mortality, and all-cause mortality.

	ESRD ^a,b^			CV Mortality ^a,b^			All-cause Mortality ^b^		
	KFRE	KFRE + Continuous CTR	KFRE + Categorical CTR	RMPM	RMPM + Continuous CTR	RMPM + Categorical CTR	RMPM	RMPM + Continuous CTR	RMPM + Categorical CTR
	HR (95% CI)	HR (95% CI)	HR (95% CI)	HR (95% CI)	HR (95% CI)	HR (95% CI)	HR (95% CI)	HR (95% CI)	HR (95% CI)
CTR (Continuous)	–	1.162 (1.054, 1.282)	–	–	1.977 (1.625, 2.406)	–	–	1.297 (1.184, 1.421)	–
CTR (Categorical)									
<0.47	–	–	Ref	–	–	Ref	–	–	Ref
0.47 ≤ ~ <0.52	–	–	1.024 (0.812, 1.291)	–	–	1.414 (0.853, 2.344)	–	–	1.171 (0.962, 1.425)
0.52 ≤ ~ <0.57	–	–	1.385 (1.114, 1.723)	–	–	1.722 (1.046, 2.835)	–	–	1.236 (1.013, 1.507)
≥ 0.57	–	–	1.369 (1.092, 1.716)	–	–	3.237 (2.016, 5.198)	–	–	1.566 (1.289, 1.903)
Age	0.970 (0.965, 0.976)	0.968 (0.963, 0.974)	0.968 (0.962, 0.973)	1.038 (1.025, 1.051)	1.028 (1.015, 1.041)	1.030 (1.017, 1.043)	1.043 (1.037, 1.049)	1.039 (1.033, 1.045)	1.040 (1.034, 1.046)
Male (ref female)	1.287 (1.117, 1.483)	1.356 (1.171, 1.570)	1.355 (1.171, 1.568)	1.167 (0.893, 1.526)	1.531 (1.158, 2.023)	1.441 (1.094, 1.898)	1.319 (1.170, 1.488)	1.461 (1.289, 1.657)	1.431 (1.263, 1.621)
Diabetes	–	–	–	1.295 (0.987, 1.700)	1.279 (0.975, 1.679)	1.263 (0.962, 1.659)	1.291 (1.142, 1.460)	1.277 (1.129, 1.444)	1.276 (1.128, 1.444)
Hypertension	–	–	–	1.926 (1.285, 2.887)	1.590 (1.057, 2.392)	1.697 (1.130, 2.548)	1.485 (1.257, 1.754)	1.386 (1.172, 1.640)	1.416 (1.197, 1.674)
Anemia	–	–	–	1.153 (0.796, 1.670)	1.059 (0.728, 1.539)	1.074 (0.739, 1.559)	1.586 (1.340, 1.877)	1.546 (1.306, 1.831)	1.552 (1.311, 1.838)
eGFR (mL/min/1.73m^2^)	0.892 (0.883, 0.900)	0.892 (0.884, 0.900)	0.891 (0.883, 0.900)	0.975 (0.966, 0.984)	0.977 (0.968, 0.986)	0.977 (0.968, 0.986)	0.983 (0.979, 0.986)	0.984 (0.980, 0.988)	0.983 (0.980, 0.987)
Pooled uPCR (per 1 log unit increase)	1.707 (1.587, 1.837)	1.687 (1.569, 1.814)	1.686 (1.567, 1.814)	–	–	–	–	–	–
AIC	10590.580	10583.000	10578.860	3267.648	3224.883	3236.761	16441.920	16412.990	16423.000
BIC	10609.590	10606.750	10612.120	3288.328	3249.010	3267.781	16472.200	16448.310	16468.420
**C-statistics** ^**c**^	0.842 (0.822, 0.863)	0.843 (0.822, 0.863)	0.843 (0.823, 0.864)	0.698 (0.656, 0.741)	0.719 (0.678, 0.761)	0.720 (0.679, 0.761)	0.693 (0.673, 0.714)	0.697 (0.676, 0.717)	0.695 (0.674, 0.716)
*p*-value comparing C-statistics	Ref	0.80	0.60	Ref	0.04	0.10	Ref	0.0005	0.22

^a^ Using cause specific model for competing risk of death.

^b^ All models use multiple imputation for missing values.

^c^ Using Uno’s method to calculate c-statistics.

Abbreviations: *AIC* Akaike’s information criterion, *BIC* Bayesian information criterion, *CI* confidence interval, *CTR* cardiothoracic ratio, *eGFR* estimated glomerular filtration rate, *HR* hazard ratio, *KFRE* Kidney Failure Risk Estimation, *uPCR* urine protein-to-creatinine ratio.

## Discussion

Our findings support the independent association of the baseline CTR and the first 2-year longitudinal CTR trajectory in patients with CKD-ND with the risk of progression to ESRD and the mortality outcomes. The critical cutoff (0.44 for dialysis, 0.49 for CV mortality, and 0.45 for all-cause mortality), defined by the cross-point of the lines of HR = 1 and the lower bound of 95% CI in the dose-response plots (Fig. 2), was lower than the widely accepted value of 0.5^4,10,12,15,35^. We observed a gradient increase in the risk of requiring dialysis, CV mortality, and all-cause mortality for CKD patients in the second to the fourth quartile of baseline CTR compared to those with a baseline CTR < 0.47. In addition, the CV mortality log-linearly increased with baseline CTR among patients with advanced CKD-ND. The consistent risk patterns for longitudinal CTR trajectories strengthen the causal inference. Despite baseline CTR significantly enhances the prognostic prediction for mortality outcomes, the predictive performance for progression to ESRD is not improved compared with the conventional KFRE.

Because a lack of automation, the statistical power of related studies has been insufficient to evaluate the prognostic value of the CTR. Use of the CTR has therefore heavily depended on individual expertise, and the cutoff of 0.5 has become a convention, leaving the CTR’s potential for serving as a rapid, low-cost, and reproducible digital marker beyond heart disease unexplored^36^. Although a CTR value of 0.42–0.50 is considered within “normal range”, our findings showed the critical threshold may be lower than the conventional cutoff of 0.5, which is consistent with some previous research^3,37^. Verifying our study results and finding an optimal cutoff based on large study samples with or without kidney disease should be an urgent research priority.

Left ventricular dilation is one of the most common causes of a CTR > 0.5 and is an independent risk factor for CVD and heart failure–related mortality^38,39^. A pooled analysis of 466 patients demonstrated moderate sensitivity and specificity of 83.3% and 45.4%, respectively, for predicting left ventricular dilation with the CTR^35^. However, among 272 patients that were hospitalized, the area under the receiver operating characteristic curve for moderate and severe left ventricular or right ventricular dysfunction based on CTRs was only 0.70, with the best cut-off of CTR 0.55 having the highest Youden index^40^. Similarly, among patients receiving hemodialysis, significant associations have been consistently observed between a high CTR and all-cause mortality^13,41,42^; nevertheless, another study discovered that the CTR did not improve the predictive value for mortality^41^. The moderate diagnostic accuracy and prognostic value of the CTR have limited its clinical utility in conventional practice.

In our study, ML streamlined CTR calculation such that we could evaluate >70,000 PA-CXRs within 24 h. As far as we are aware, this is the first report of linear dose-response associations of the CTR with CV and all-cause mortality among patients with CKD-ND. We discovered that this strong association significantly improved the predictive performance of the conventional models for mortality risk assessment in CKD^21^. However, the CTR only marginally improved the predictive power of the KFRE among patients with CKD. Indeed, the effect size of HR does not lend itself to drawing direct conclusions regarding the contribution of the CTR to prognosis prediction^43^. Varga et al. has systematically reviewed this issue recently^44^. The reason underlying the discrepancy of the significantly positive association but poor predictive capability of the CTR with all outcomes of interest may be explained by the follows: (1) the predictive variables of KFRE or RMPM have predicted the outcomes of ESRD and mortality well and the predictive role of CTR is therefore not standing out; (2) there were large overlapping distributions of CTR when stratifying the population based on the outcomes of interest (Supplementary Fig. 5); (3) CTR is a summarized and relatively low-resolution digital cardiac marker measuring a simple ratio between cardiac and thoracic horizontal diameter on the PA-CXR, rather than providing detailed information about anatomical abnormalities such as left ventricular mass index or left atrial volume index and functional impairments such as systolic or diastolic dysfunction. Therefore, it may be insufficient to provide additional prognostic benefit on top of the original KFRE or RMPM models. Nevertheless, our findings encourage future studies to identify ML-driven cardiac digital markers that are more disease prognosis specific to the development of ESRD and mortality, particularly using common clinical imaging modalities to infer important anatomical and functional features.

Our results suggest that CTR can be used to personalize CKD care; this suggestion is also supported by the observations of a large epidemiological study in which ~50% of patients with CKD-ND experienced CV mortality^45^. Although cardiorenal syndrome may explain this phenomenon^46^, the exact pathogenesis remains unclear. A recent animal study revealed that the monocytic expression of G protein-coupled receptor 68 (GPR68) triggered by retinol and its binding protein (RBP4) exacerbates CKD-induced inflammation and fibrosis of the heart^47^. In human, single-nucleotide polymorphisms of RBP4 have been associated with obesity, diabetes, and, particularly, cardiovascular disease^48^. Chronic myocardial inflammation has been reported to involve in the pathophysiological process of cardiomegaly and remodeling^49^. In addition, low calcitriol levels among patients with CKD may be associated with decreases in the number and functions of peripheral endothelial progenitor cells (EPCs), which are linked to CVD^50^. It is also reported the dysfunction of EPCs may associate with adverse cardiac remodeling and impaired myocardial perfusion, leading to heart failure^51^. To fully understand cardiorenal interactions throughout the entire course of CKD, regular and systematic evaluation of heart function among patients with CKD is essential. Research should focus on optimizing heart functional evaluation and clarifying the detrimental role of cardiac dysfunction in different CKD stages^52^. As the healthcare industry has entered the era of digital transformation and innovation, integrating ML powered annotation into the existing healthcare paradigms is essential to achieve universal automated disease screening services and harness the potential to significantly improve the healthcare efficiency and equity. In the current practice, a CXR image does not provide an automated measurement of CTR, not to mention the prediction of future risk probability ranging from CKD progression, CVD, to all-cause mortality and the design of actionable targets. This is exactly the reason why CTR is currently underused in the clinical practice. If the clinical effectiveness through the CTR-driven cardiorenal care bundle can be confirmed, CTR will likely become a routine digital marker in CKD care.

The findings of vulnerabilities of increasing CTR among patients without hypertension deserved attention. This phenotype, cardiomegaly without a hypertension history, represents a unique clinical challenge as the opportunity to optimize heart size is limited. It is also likely that hypertension-free patients with significant cardiomegaly and CKD may exhibit hemodynamic instability and compromise the kidney perfusion that accelerates the progression to ESRD. Given scarce evidence, more research efforts are warranted to confirm our findings and establish the pathogenic basis underlying this phenomenon.

The strengths of this study include its high-quality data collection methods, which comprise longitudinal measurement of the CTR, a large sample size, and rigorous and systematic statistical and ML analyses. The study also has several limitations. A lack of external validation limits the generalizability of the findings from this single-center study. Furthermore, we encountered potential confounding, with the decision to undergo CXRs being associated with the patient characteristics that influenced outcomes. However, in subgroup analysis, a greater risk of developing dialysis was observed among patients without CVD or hypertension; with respect to mortality outcomes, the prognostic value of the CTR was not affected by CVD status. This study is further limited by the possibility of residual confounding (eg, physical activity, environmental exposure, and heritability information)^53^ and overadjustment for variables that may lie along the causal pathway (eg, CVD). Last but not least, the distribution of CTR may be differential between men and women and therefore using sex-specific cutoffs of CTR may help improve the accuracy of risk assessment. However, due to the reduced sample size in the sex-stratified analysis, it would be difficult to provide robust evaluation of appropriate sex-specific cutoff values of CTR for clinical practice in the present study.

## Conclusions

This study demonstrated the prognostic value of the CTR in patients with CKD by using an ML-powered annotation tool. Validation of the predictive performance and clinical effectiveness of the CTR in multi-center clinical trials for progression to ESRD and mortality among patients with CKD-ND warrants adoption of this ML application in clinical CKD care. Nevertheless, our research presents a methodological foundation for using ML to improve cardioprotection among patients with CKD.

## Supplementary information

Supplementary Data 1

Supplementary Data 2

Supplementary Data 3

Supplementary Data 4

Supplementary Data 5

Supplementary Data 6

Supplementary Material

Description of Additional Supplementary Files

Reporting Summary

## Data Availability

The data that support the findings of this study are available on request from the corresponding author, CCK. The data are not publicly available due to them containing information that could compromise research participant privacy. Addition data of dose-response plots are provided at https://figshare.com/articles/dataset/Dose_response_xlsx/21679154.

## Code availability

Code for prediction model development is provided at https://zenodo.org/record/7403096#.Y47s0HZByUk^54^. Further detail of all the code developed during the study is available from chinchik@gmail.com on reasonable request.
