# Supplementary material for: Cardiothoracic ratio values and trajectories are associated with risk of requiring dialysis and mortality in chronic kidney disease
Source: Commun Med (Lond). 2023 Feb 7;3:19. doi: 10.1038/s43856-023-00241-9 (PMC9905092; doi:10.1038/s43856-023-00241-9)

Supplementary Material

Supplementary Table 1. Frequency of CTR measurement in the study population

Supplementary Table 2. The Characteristics of Selected Echocardiographic Parameters by Baseline CTR Quartile

Supplementary Figure 1. Model Architecture of U-Net for the CTR Estimation Algorithm

Supplementary Figure 2. ML-Based CTR estimation Based on Images From Each Baseline CTR Quartile

Supplementary Figure 3. Trajectory Group of CTR by LCMM

Supplementary Figure 4. Calibration Plot of Predicted and Observed Probability of (a) Progression to ESRD, (b) CV Mortality, and (c) All-cause Mortality

Supplementary Figure 5. Comparison of Distribution Curve for Baseline CTR for Outcomes of Interest: (a) Progression to ESRD, (b) CV Mortality, and (c) All-cause Mortality

Supplementary Table 1. Frequency of CTR measurement in the study population

No. of CTR measurement	No. of patients	Percentage (%)
2	656	26.52
3	488	19.73
4	361	14.59
5	254	10.27
6	156	6.31
7	134	5.42
8	116	4.69
9	70	2.83
≥ 10	239	9.64
Total	2474	100.00

Supplementary Table 2. The Characteristics of Selected Echocardiographic Parameters by Baseline CTR Quartile

Characteristics	N (%)	Total	Baseline CTR (Quartile of baseline period)				p-value
			CTR < 0.47	0.47 ≤ CTR < 0.52	0.52 ≤ CTR < 0.57	CTR ≥ 0.57	
Echocardiographic parameters [a]							
Baseline population	3117 (100.0)						
Ejection fraction (%), median (IQR)	1193 (38.3)	59.6 (53.4, 65.0)	59.9 (55.2, 65.0)	59.5 (54.3, 65.3)	60.9 (53.9, 65.3)	58.4 (50.4, 64.3)	<0.01
Ejection fraction < 45 %, n (%)	1193 (38.3)	148 (12.4)	16 (9.8)	18 (7.2)	35 (10.8)	79 (17.4)	<0.01
LVMI (g/m^2) , median (IQR)	1161 (37.2)	95.6 (79.6, 115.7)	85.8 (68.7, 103.7)	88.6 (73.3, 107.0)	94.7 (78.8, 114.9)	103.7 (86.1, 124.0)	<0.01
LAVI (mL/m^2) , median (IQR)	1136 (36.4)	34.0 (25.5, 44.3)	26.8 (21.2, 35.5)	30.6 (22.2, 38.5)	34.7 (26.3, 44.2)	39.3 (31.2, 50.0)	<0.01
Trajectory population	2474 (100.0)						
Ejection fraction (%), median (IQR)	1034 (41.8)	59.4 (52.8, 64.9)	59.5 (55.1, 64)	59.4 (54.3, 64.9)	60.9 (53.9, 65.4)	58.0 (50.0, 64.5)	<0.01
Ejection fraction < 45 %, n (%)	1034 (41.8)	134 (13.0)	15 (10.2)	16 (7.3)	31 (11.2)	72 (18.4)	<0.01
LVMI (g/m^2), median (IQR)	1006 (40.7)	95.0 (79.3, 115.1)	85.8 (68.7, 102)	88.2 (72.8, 106.6)	94.9 (78.8, 113.5)	103.8 (85.9, 123.8)	<0.01
LAVI (mL/m^2) , median (IQR)	983 (39.7)	34.1 (25.6, 44.8)	26.9 (21.5, 36.3)	30.0 (22.1, 38.6)	35.4 (26.7, 45.0)	39.3 (31.2, 50.3)	<0.01

[a] Echocardiographic parameters measurements that were obtained within -1 year to +1 year and closest to the index date.

Abbreviations: CTR, cardiothoracic ratio; IQR, interquartile range; LVMI, left ventricular mass index; LAVI, left atrial volume index.

Supplementary Figure 1. Model Architecture of U-Net for the CTR Estimation Algorithm

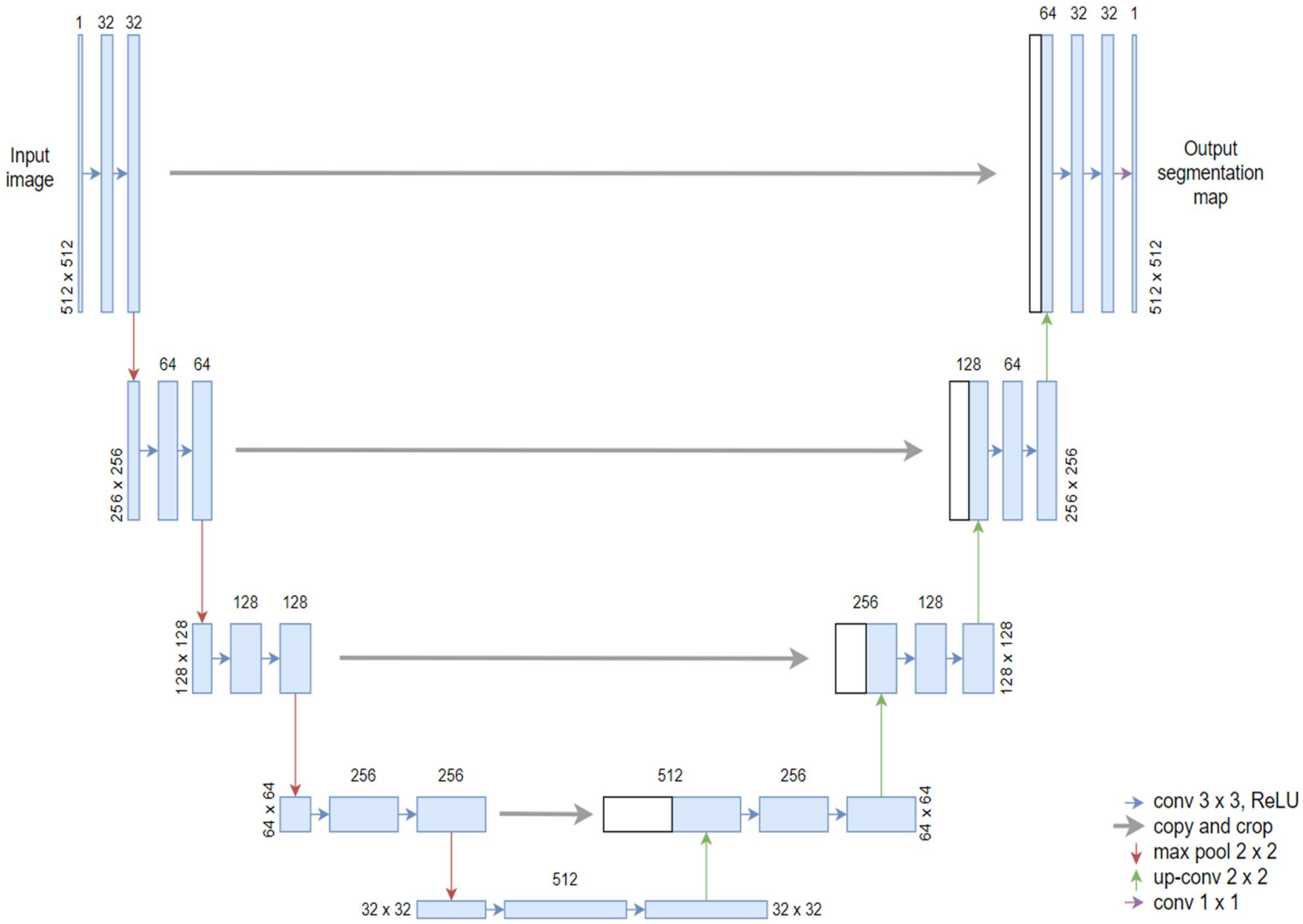

Supplementary Figure 2. ML-Based CTR estimation Based on Images From Each Baseline CTR Quartile

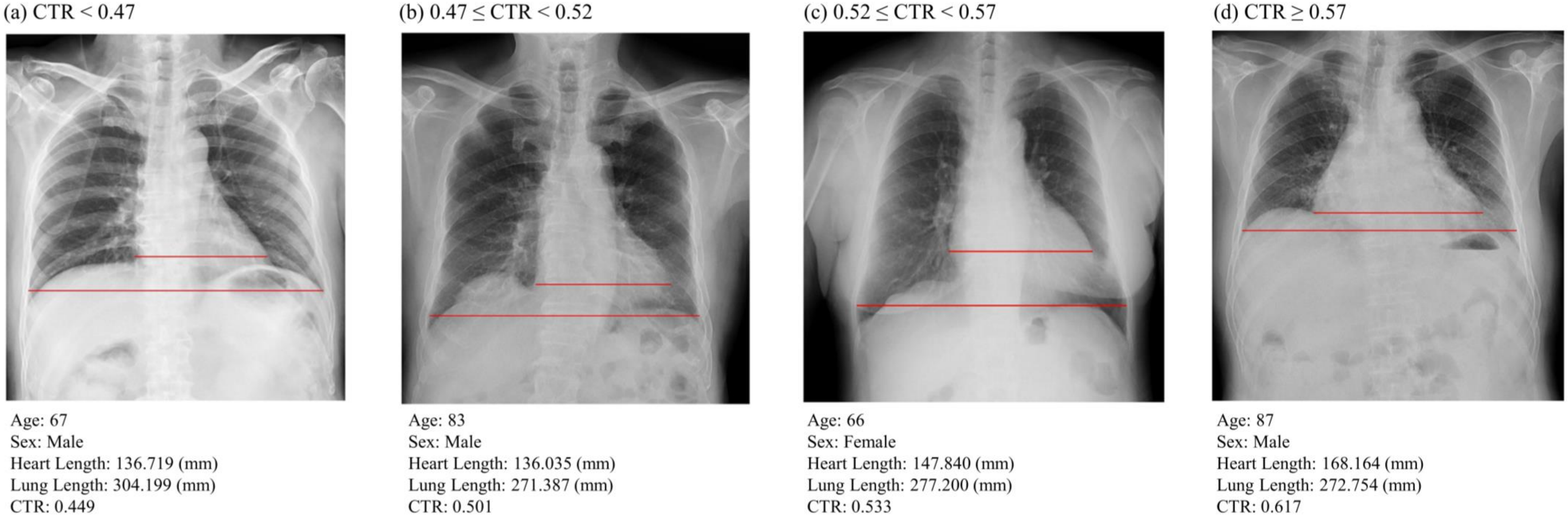

Supplementary Figure 3. Trajectory Group of CTR by LCMM

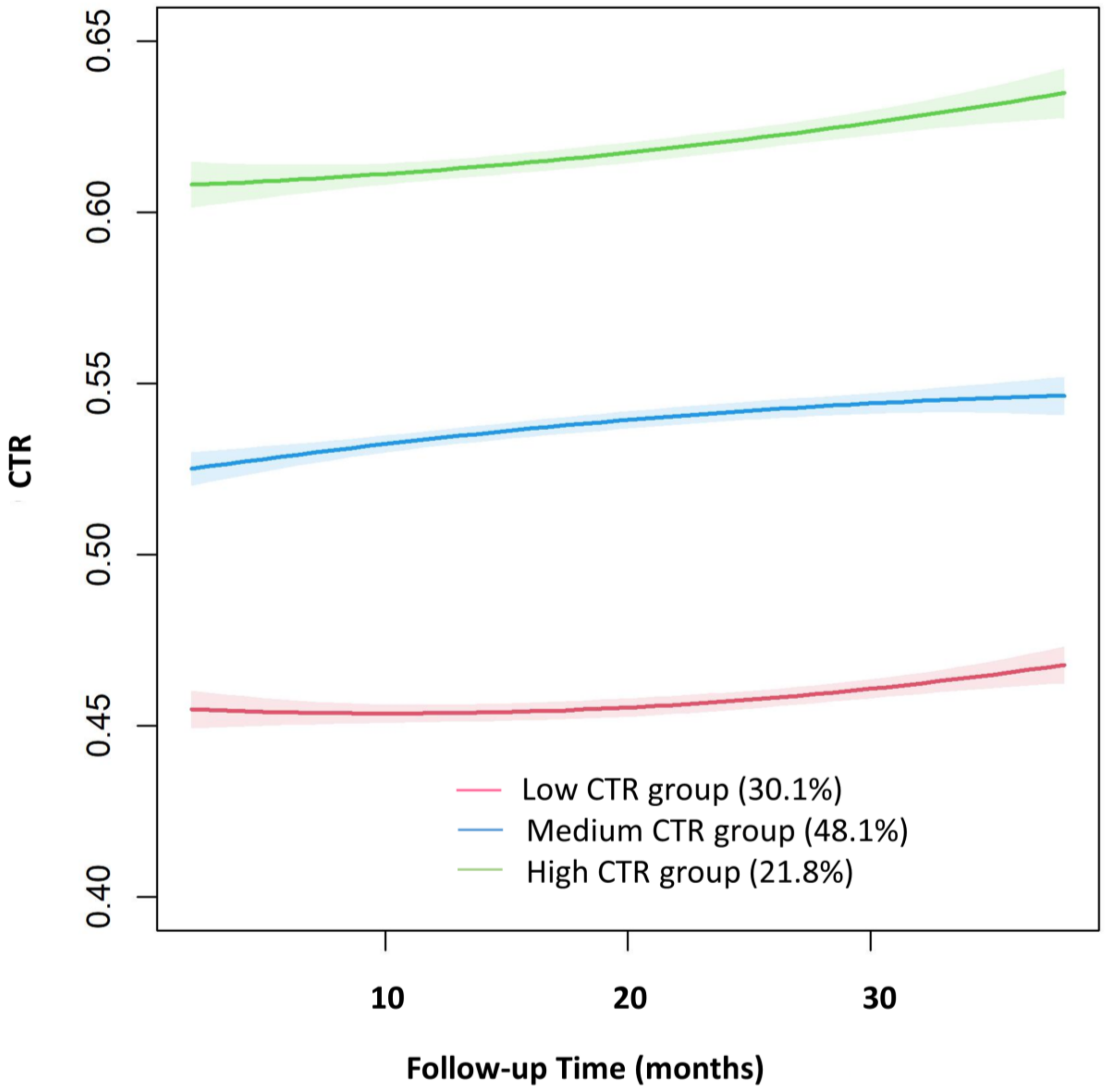

Supplementary Figure 4. Calibration Plot of Predicted and Observed Probability of (a) Progression to ESRD, (b) CV Mortality, and (c) All-cause Mortality

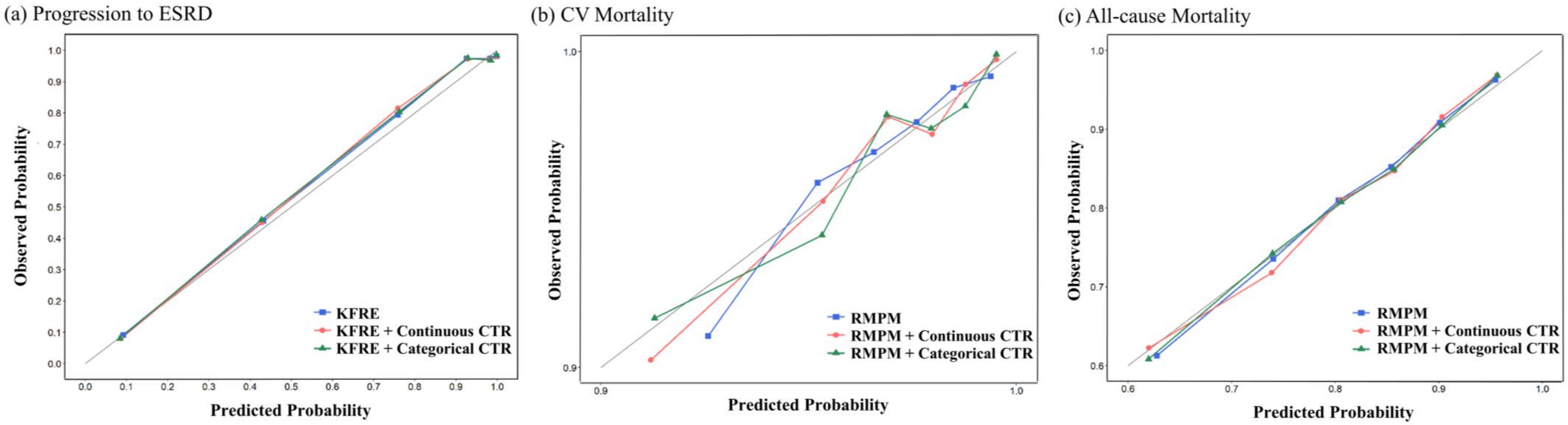

Supplementary Figure 5. Comparison of Distribution Curve for Baseline CTR for Outcomes of Interest: (a) Progression to ESRD, (b) CV Mortality, and (c) All-cause Mortality

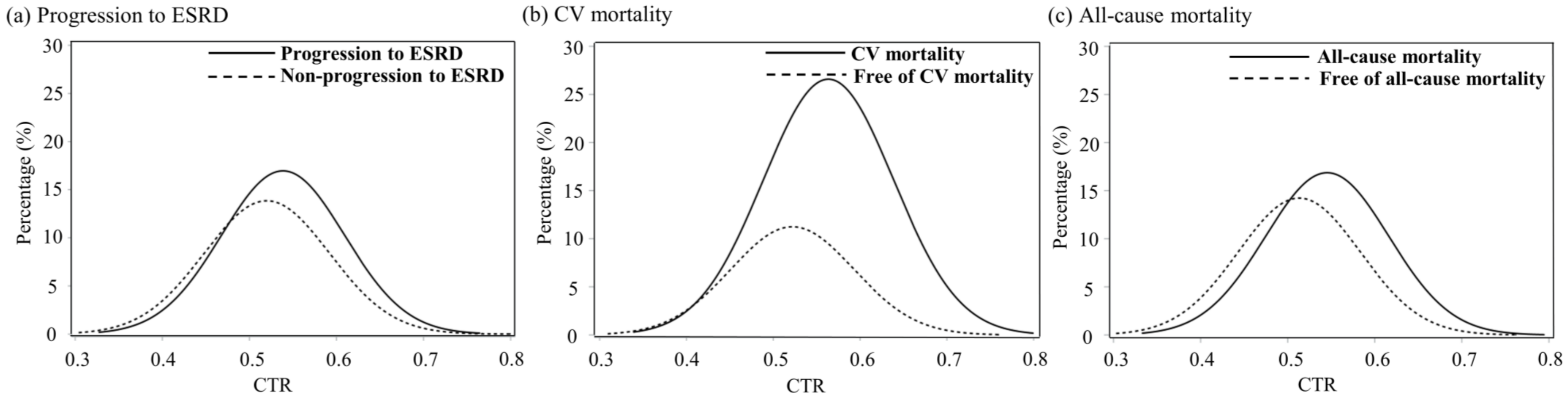

Supplement: Supplementary file 7 — Supplementary Material [file 43856_2023_241_MOESM7_ESM.pdf]
