# Supplementary material for: Cardiothoracic ratio values and trajectories are associated with risk of requiring dialysis and mortality in chronic kidney disease
Source: Commun Med (Lond). 2023 Feb 7;3:19. doi: 10.1038/s43856-023-00241-9 (PMC9905092; doi:10.1038/s43856-023-00241-9)
Supplement: Supplementary file 8 — Description of Additional Supplementary Files [file 43856_2023_241_MOESM8_ESM.pdf]

Description of Additional Supplementary Files

File Name: Supplementary Data 1

Description: Biochemical and Medication Profiles by Baseline CTR Quartile

File Name: Supplementary Data 2

Description: Demographic and Clinical Characteristics by Baseline CTR Quartile

File Name: Supplementary Data 3

Description: Demographic and Clinical Characteristics by Trajectory Groups Derived by LCMM

File Name: Supplementary Data 4

Description: HRs (95% CIs) for Progression to ESRD, CV Mortality, and All-Cause Mortality by Baseline CTR (N = 3117) Quartile and CTR Trajectory (N = 2474) Group

File Name: Supplementary Data 5

Description: Adjusted HRs (95% CIs) for Progression to ESRD, CV Mortality, and All-cause Mortality According to Baseline CTR Stratified by Age, Sex, Hypertension, Diabetes, CVD, CKD Stage and Proteinuria

File Name: Supplementary Data 6

Description: Adjusted HRs (95% CIs) for Progression to ESRD, CV Mortality, and All-cause Mortality According to CTR Trajectory Group Stratified by Age, Sex, Hypertension, Diabetes, CVD, CKD Stage and Proteinuria
